# Cure of Leprosy

**Published:** 1890-02-08

**Authors:** 


### CURE OF LEPROSY.

Dr. Philippo, of Kingston, Jamaica, has reported a case of leprosy completely cured by the use of gurjun and chaulmoogra oils, or, at any rate, by the latter. The patient cut his foot while bathing in the sea in 1872, the wound was slow in healing, re-opening with swelling of the toe at intervals for several months. In 1874 he noticed a scaly eruption around the ankle, which in 1876 became general, the hands swelling, and next the ears and face, other symptoms of leprosy following. In 1879 he was unable to walk, when towards the end of that year Dr. Philippo took him in hand. The gurjun oil, with lime water, was rubbed in all over the body twice a day, the patient bathing before each application, though the treatment had often to be intermitted owing to attacks of fever. It was also taken internally until 1881, when chaulmoogra oil was substituted for internal use. Gradual improvement then set in, beginning in the face. In 1884 he was able to walk a little, in 1885 had quite recovered the use of his legs, and early in 1886 he was pronounced cured and discontinued the oils. "From that time," wrote the patient, "I ceased to live in grease," and he has now remained for four years free from the least symptom of the disease.

Dr. Philippo, who has had considerable acquaintance with leprosy, is strongly opposed to the notion that it is in any way connected with the use of a fish diet, a theory which is not borne out by the experience of some of the best observers. He admits that leprosy is in Jamaica more frequently seen on the coast than in the interior, but considers that this fact is largely due to the habit of the poorer class of lepers of flocking to the towns, which are there, as elsewhere, mostly near the sea, for the charity to be obtained in the larger centres of population. He firmly believes in its hereditary and contagious character, and is inclined to look on it as allied to tuberculosis. With regard to the treatment by gurjun and chaulmoogra oils, he admits that the results have disappointed the expectations at first raised by its advocates, but attributes the unequivocal success achieved in this particular case to the thoroughness and perseverance with which it was carried out for six years, and Dr. Phineas Abraham, to whom we are indebted for this communication, agrees with Dr. Philippo, that the reported failures are in many cases due to the insufficient employment of the oils, both as regards quantity and duration. It is only, he says, in properly-appointed special hospitals or asylums that the necessary conditions for such prolonged and troublesome a method of treatment can be secured; but whatever difficulties may surround the general practitioner, the success obtained by Dr. Philippo should encourage medical men and their patients to persevere.

